# Risk of emergency hospital admission in children associated with mental disorders and alcohol misuse in the household: an electronic birth cohort study

**DOI:** 10.1016/S2468-2667(18)30069-0

**Published:** 2018-06-05

**Authors:** Shantini Paranjothy, Annette Evans, Amrita Bandyopadhyay, David Fone, Behnaz Schofield, Ann John, Mark A Bellis, Ronan A Lyons, Daniel Farewell, Sara Jayne Long

**Affiliations:** a Division of Population Medicine, School of Medicine, Cardiff University, Cardiff, UK; b Centre for the Development and Evaluation of Complex Interventions for Public Health, School of Social Sciences, Cardiff University, Cardiff, UK; c Centre for Improvement in Population Health Through E-records Research, Swansea University, Swansea, UK; d Public Health Wales NHS Trust, Cardiff, UK

## Abstract

**Background:**

Mental disorders and alcohol misuse are common in families but their effects on the physical health of children are not known. We investigated the risk of emergency hospital admissions during childhood associated with living with an adult who has a mental health disorder, or who had an alcohol-related hospital admission.

**Methods:**

We did this cohort study in a total population electronic child cohort in Wales, UK, which includes all children who live in Wales or with a mother who is resident in Wales. We used Cox regression to model time to first emergency hospital admission during the first 14 years of life associated with living with an adult who has a mental health disorder, or who had an alcohol-related hospital admission. We adjusted our results for social deprivation and perinatal risk factors.

**Findings:**

We included data for 253 717 children with 1 015 614 child-years of follow-up. Living with an adult with a mental disorder was associated with an increased risk of emergency admission for all causes (adjusted hazard ratio [aHR] 1·17, 95% CI 1·16–1·19), for injuries and external causes (1·14, 1·11–1·18), and childhood victimisation (1·55, 1·44–1·67). Children living with a household member who had an alcohol-related hospital admission had a significantly higher risk of emergency admissions for injuries and external causes (aHR 1·13, 95% CI 1·01–1 ·26) and victimisation (1·39, 1·00–1·94), but not for all-cause emergency admissions (1·01, 0·93–1·09).

**Interpretation:**

The increased risk of emergency admissions in children associated with mental disorders and alcohol misuse in the household supports the need for policy measures to provide support to families that are affected.

**Funding:**

Economic and Social Research Council, Medical Research Council, Alcohol Research UK, Public Health Wales.

## Introduction

Health, social, and economic outcomes during life are heavily influenced by biological, social, and environmental exposures during pregnancy and childhood. Exposure to adverse childhood experiences, including mental illness and alcohol misuse in the family, is associated in adulthood with substance misuse, mental ill-health, obesity, heart disease, cancer, unemployment, and involvement in violence.^1^ In the UK, severe mental illnesses such as schizophrenia and bipolar disorder affect 1–2% of adults^2^, ^3^ but common mental disorders, including depression, anxiety, panic, and somatisation, can affect 16% of adults.^4^ Alcohol misuse is also prevalent, with 6·6% of adults consuming hazardous levels of alcohol and a further 2–3% categorised as dependent drinkers. A significant proportion in both categories also have mental disorders.^5^ The proportion of children who are exposed to mental illness or alcohol misuse is not known, although it is estimated that up to 30% of children live with an adult binge drinker.^6^ Little is known about how children are affected by patterns of alcohol consumption, including drinking at low risk levels and heavy episodic or binge drinking.^7^

Mental illness and alcohol misuse in families is associated with relationship conflict and can lead to disruption of routines, unpredictable parenting, and inconsistent care.^8^, ^9^ Parental mental disorders and alcohol misuse are associated with substance misuse, behavioural problems, and impaired mental health in children^7^, ^10^ but few studies have investigated the association with physical health outcomes during childhood.^7^ Inconsistent care can lead to increased susceptibility to infections^11^, ^12^ and injuries in children. Prenatal alcohol exposure is associated with fetal alcohol spectrum disorders and can also impair early lung development, increasing susceptibility to respiratory illnesses during childhood.^13^ Emergency hospital admissions are unpredictable, occur at short notice as a result of clinical need, and are common in children, primarily because of respiratory infections.^14^ Emergency admissions because of childhood victimisation (maltreatment-related injuries, where there is concern about the welfare of the child),^15^ injuries, and external causes (eg, accidents, self-harm, and assault) might be associated with inconsistent emotional or physical care, which might in turn be related to mental disorders or alcohol misuse in the household.

**Research in context**

**Evidence before this study**

We searched PubMed from the year 2000 with the terms “adverse childhood experiences*”, “impact of childhood experiences on health*”, (adverse childhood experiences AND mental health) OR (adverse childhood experiences AND parental alcohol use) OR (adverse childhood experiences AND childhood health) OR (‘adult mental health OR family mental health OR parental mental health OR parental drinking OR family drinking OR adult drinking’ AND child health AND cohort studies). Titles and abstracts were reviewed and we identified eight reviews and 32 primary studies that were relevant.

Most studies on the effects of adverse childhood experiences were cross-sectional or retrospective cohort studies that investigated the association with adult physical and mental health outcomes, or outcomes in early adulthood (age 18–20 years). Three studies examined early experience of adversity and outcomes during childhood; two of these were in children at high risk (ie, with reported or at risk of maltreatment, or receiving child welfare) and one prospective cohort analysis showed an association between exposure to adverse events before age 8 years and inflammatory markers (interleukin-6 and C-reactive protein) at age 10 years. Mental disorders and alcohol misuse in parents can affect the provision of care and the physical health of children—eg, increased susceptibility to infections because of under-nourishment or poor hygiene or injuries due to lack of adult supervision or physical harm due to neglect. Most studies to date have studied associations with mental health and substance misuse outcomes in children, so the effect of these exposures on physical heath during childhood is not known.

**Added value of this study**

To our knowledge, our study is the first total population cohort study that investigates the association between living with an adult who has a mental disorder or who had an alcohol-related hospital admission and emergency hospital admission. The use of routinely available health-care data enabled the objective measurement of these exposures, thus overcoming some of the limitations associated with reporting and same source bias in previous studies of adverse childhood experiences. We showed an increased risk of emergency admissions in children associated with household mental disorders and alcohol use, which was independent of family structure and area level deprivation.

**Implications of all the available evidence**

Socioeconomic conditions and other factors that imply vulnerability in families such as young maternal age, exposure to mental disorders, and alcohol misuse cumulatively contribute to the risk of emergency hospital admissions in children. Pregnant women and children exposed to mental disorders or alcohol-related problems in the family can potentially be identified when they contact health services. Policy measures are needed to address socioeconomic conditions and support families with mental disorders or alcohol-related problems who have or are planning to have children. Further research is needed to explore the effect of these exposures on child development and education.

The availability of record-linked routine administrative and health-care datasets for research provides the opportunity for total population-based analyses of a wide range of social and environmental factors and their association with child health and education outcomes. Adverse childhood experiences have not yet been coded in UK administrative data. We previously developed algorithms for coding mental disorders^16^, ^17^ and alcohol-related hospital admissions^18^ using these datasets. In this study, we aim to use our previous work to code for these exposures and investigate the risk of emergency hospital admission during childhood (due to all causes, external causes and injury, and victimisation) associated with living with an adult who has a mental health disorder, or with an adult who has a significant alcohol-related illness. We also explored whether or not these associations varied according to the timing of exposure, and the effect of the combination of these two exposures, together with markers for family structure such as young maternal age, living in a single adult household, and area-level social deprivation.

## Methods

### Data sources and study design

The Wales Electronic Cohort for Children includes 981 404 children born between Jan 1, 1990, and Oct 7, 2012, with a mother or child resident in Wales.^19^ Eligible participants were identified from the Wales Demographic Service Dataset, an administrative register of all individuals living in Wales registered with a general medical practitioner (GP). The Wales Electronic Cohort for Children is derived from record-linkage of de-identified routinely collected data held in health and social datasets and made available to the privacy-protecting Secure Anonymised Information Linkage (SAIL) databank at Swansea University, UK.^20^, ^21^ For each dataset within the SAIL databank, individuals are assigned a unique Anonymised Linking Field based on encrypted National Health Service numbers provided by NHS Wales Informatics Service. The SAIL linkage system uses a combination of deterministic (based on NHS numbers) and probabilistic record linkage (based on first name, surname, date of birth, gender, and phonex and soundex version of names).^21^ The linkage is more than 99·85% accurate.^21^ Second stage encryption is used by the databank before storing data and third stage encryption is used to create project-specific datasets and linkage between datasets. Residential anonymised linking fields (RALFs) are created by encrypting addresses within NHS Wales Informatics Service.^22^, ^23^ Linkage to the encrypted anonymised linking fields enables individuals living in the same household to be anonymously linked and each child was assigned a RALF for each address during the study period. We used record-linked data from the databases shown in the appendix (p 1), for Jan 5, 1998, to Oct 7, 2012, defined by the availability of data on hospital admissions from the Patient Episode Database for Wales.

We included livebirths in Wales for which there was a valid RALF and sufficient data on primary care consultations for adult household members in the SAIL databank to enable ascertainment of exposure groups (figure). Data were censored for migration out of Wales (identified from the Wales Demographic Service), death during infancy or childhood (identified from the Public Health mortality files), and first emergency hospital admission, or for cessation of exposure data.

**Figure:**
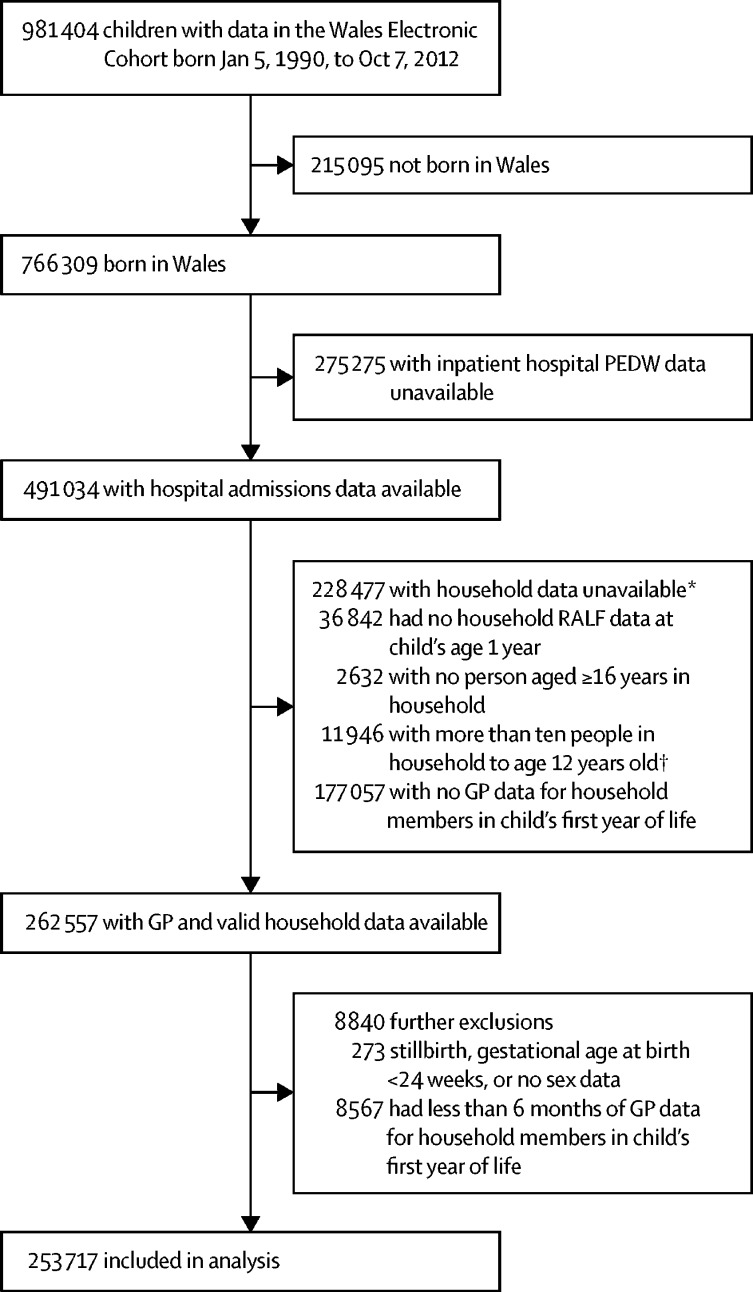
Participant selection GP=general practitioner. RALF=residential anonymised linking field. PEDW=Patient Episode Database Wales. *Except for children who died (n=1686) or moved out of Wales (n=5020) in the first year of life. †The Unique Property Reference Number is considered inaccurate if there are more than ten people in a household.

The Wales Electronic Cohort for Children was approved by an independent Information Governance Review Panel. The Research Ethics Committee for Wales judged the Wales Electronic Cohort for Children to be an anonymised research database that does not require ethical review, in line with National Ethics Committee guidance.

### Exposures and outcomes

We defined two measures of adverse childhood experiences: first, living with an adult with a current or historical mental disorder (anxiety, depression, bipolar, or schizophrenia);^16^, ^17^ and second, living with an adult who had an alcohol-related emergency hospital admission during the exposure periods.^18^

We had previously developed and validated a common mental disorder algorithm using a set of Read code lists (diagnosis, symptoms, treatments) for the identification of anxiety and depression in the General Practice Database within the SAIL databank, and showed that the incorporation of symptom codes (to reflect GP coding behaviours) provides a high specificity and positive predictive value of detecting cases of anxiety and depression from routine GP data.^16^ We used this algorithm in combination with another validated algorithm that was designed to search general practice data for patients with a lifetime diagnosis of psychotic disorders within the SAIL databank.^17^

We defined four exposure periods: birth to <1 year, 1–<5 years, 5–<8 years, and 8–<12 years. We defined current exposure from a search for relevant Read codes during each exposure period for any adult that was living with the child on their 1st, 5th, 8th, and 12th birthday. We used this approach to capture people who may present to their GP with a common mental disorder but not be diagnosed for a period of time and also those who may delay seeing their GPs for a period of time. We assigned a date of exposure that corresponded with the end date in each exposure period, so that the analysis could take account of the temporal relationship between exposure and outcome. We defined historical exposure from a search for relevant Read codes for any adult living with the child through the retrospective longitudinal data in the General Practice Database before the child was born. The period for which retrospective data were available varied between individuals depending on the length of their registration with a practice that supplies data to the Secure Anonymised Information Linkage dataset (median 5·65 years, IQR 2·61–9·79). We followed the same process to ascertain adults who had an alcohol-related hospital admission using codes we had defined previously.^18^

The primary outcome measures were the first emergency hospital admissions recorded in Patient Episode Database for Wales for (1) all causes, (2) injuries and external causes (ICD-10 codes S00–T19, T20–T32, T33–T35, T36–T71, T74, T75, T79, V01–V99, W00–X59, X60–X84, X85–Y09, Y10–Y36 in any coding position), and (3) childhood victimisation

### Statistical analysis

We used Cox regression with discrete time-varying exposure covariates (0 until first exposure, 1 thereafter) to model time to the first all-cause emergency hospital admission in the first 14·7 years of life. We estimated hazard ratios (HRs) with 95% confidence intervals (CI) for each exposure, adjusted for confounding variables. We tested the inclusion of two-way interaction terms between both exposures, and between each exposure and maternal age, small-area deprivation, single adult household, and timing of exposure, using likelihood ratio tests. None of these interactions, with the exception of the interaction between household mental health and timing of exposure, improved the fit of the model to the data.

We repeated the analyses to estimate HRs for emergency hospital admission because of injuries and external causes and childhood victimisation, adjusted for maternal and perinatal characteristics. We also repeated these analyses using the Andersen-Gill model to explore the effect of including all emergency admissions for each child during the follow-up period.

We constructed a Direct Acyclic Graph^24^ to inform our choice of variables to include in the analysis (appendix p 2). We adjusted for small-area deprivation (based on Townsend score,^25^ calculated with data from the 2001 census), young maternal age (<18 years), and living in a single adult household because they are potential confounders. Perinatal factors (maternal smoking during pregnancy, gestational age, and breastfeeding at birth or 6–8 weeks [when the NHS collects such data]) could be confounders or be considered to be on the causal pathway. As we were interested in the effects of the exposures that were not mediated through these factors, we adjusted for these in the model.

Data were missing for breastfeeding for 14·7% of records and for maternal smoking for 65·6% of records in the dataset, due to organisational and administrative differences in data collation between hospitals in Wales, suggesting that these data can be reasonably assumed to be missing at random. The subset for which data were available was large enough to fit an imputation model for these covariates with sufficient precision. The proportion of missing data for all other variables was less than 5%. We used multiple imputation with chained equations^26^ to account for missing data under the missing at random assumption. The imputation model included all co-variates, an event indicator, and the cumulative baseline hazard as described by White and Royston.^27^ Results from the Cox regression models using complete cases and the imputed dataset were consistent (appendix p 4). We have therefore presented results from the multiply imputed datasets.

We used Stata IC (version 13) for statistical analyses.

### Role of the funding source

The funders had no role in designing the study, data collection, analysis, or interpretation, or in writing the report. MAB's role in the design, analysis, and writing was independent of the funding from Public Health Wales. The corresponding author had full access to all the data in the study and final responsibility for the decision to submit for publication.

## Results

253 717 eligible children born in Wales between 1998 and 2012, listed in the Wales Electronic Cohort for Children were included (figure 1). The total length of follow-up was 1 015 614 child-years (median 2·48 years; IQR 0·75–6·46). Sociodemographic characteristics of the cohort are consistent with national population statistics for these variables (table 1, appendix p 3). Exposure to household mental disorders, exposure to alcohol-related hospital admissions, and emergency hospital admission were higher in more deprived areas (table 1). Overall, 6985 (2·8%) of children were born to a mother younger than age 18 years and 54 046 (21·9%) of infants lived in a single adult household (table 1). By age 11 years, 15 636 (43·7%) children had lived in a single adult household at some point.

**Table 1:** Sociodemographic and perinatal characteristics in each exposure and outcome group

	**Total (n=253 717)**	**Ever lived with household members with mental health disorder (n=116 177)**	**Ever lived with household members with alcohol-related hospital admission (n=9499)**	**Ever had an all-cause emergency hospital admission (n=128 434)**	**Ever had an emergency external cause or injury hospital admission (n=23 345)**	**Ever had a victimisation hospital admission (n=3198)**
**Townsend deprivation quintile at birth or in first 4 months**						
1 (least deprived)	42 019 (16·6%)	17 649 (15·2%)	885 (9·3%)	19 707 (15·3%)	3146 (13·5%)	251 (7·8%)
2	44 994 (17·7%)	19 974 (17·2%)	1154 (12·1%)	21 699 (16·9%)	3808 (16·3%)	450 (14·1%)
3	49 403 (19·5%)	22 091 (19·0%)	1691 (17·8%)	25 105 (19·5%)	4491 (19·2%)	519 (16·2%)
4	53 368 (21·1%)	25 597 (22·0%)	2254 (23·7%)	27 592 (21·5%)	5011 (21·5%)	665 (20·8%)
5 (most deprived)	63 411 (25·0%)	30 706 (26·4%)	3492 (36·8%)	34 127 (26·6%)	6838 (29·3%)	1306 (40·8%)
Missing data	522 (0·2%)	160 (0·1%)	23 (0·2%)	204 (0·2%)	51 (0·2%)	7 (0·2%)
**Sex**						
Male	130 421 (51·4%)	59 724 (51·4%)	4851 (51·1%)	70 380 (54·8%)	13 256 (56·8%)	1705 (53·3%)
Female	123 296 (48·6%)	56 453 (48·6%)	4648 (48·9%)	58 054 (45·2%)	10 089 (43·2%)	1493 (46·7%)
**Breastfeeding at birth or at 6–8 weeks**						
No	97 730 (38·5%)	50 562 (43·5%)	4531 (47·7%)	52 491 (40·9%)	9813 (42·0%)	1608 (50·3%)
Yes	118 651 (46·8%)	49 507 (42·6%)	3177 (33·4%)	56 867 (44·3%)	9491 (40·7%)	1098 (34·3%)
Missing data	37 336 (14·7%)	16 108 (13·9%)	1791 (18·9%)	19 076 (14·9%)	4041 (17·3%)	492 (15·4%)
**Maternal age at childbirth**						
<18 years	6985 (2·8%)	3692 (3·2%)	550 (5·8%)	4219 (3·3%)	1056 (4·5%)	219 (6·8%)
18–24 years	72 363 (28·5%)	36 155 (31·1%)	3650 (38·4%)	40 483 (31·5%)	8211 (35·2%)	1371 (42·9%)
25–29 years	70 161 (27·7%)	31 677 (27·3%)	2291 (24·1%)	35 420 (27·6%)	6154 (26·4%)	742 (23·2%)
30–34 years	65 455 (25·8%)	27 946 (24·1%)	1878 (19·8%)	30 737 (23·9%)	5056 (21·7%)	527 (16·5%)
≥35 years	38 629 (15·2%)	16 660 (14·3%)	1125 (11·8%)	17 518 (13·6%)	2857 (12·2%)	329 (10·3%)
Missing data	124 (0%)	47 (0%)	5 (0·1%)	57 (0%)	11 (0%)	10 (0·3%)
**Gestational age at birth**						
24–<28 weeks	1063 (0·4%)	311 (0·3%)	35 (0·4%)	547 (0·4%)	57 (0·2%)	24 (0·8%)
28–<33 weeks	3347 (1·3%)	1600 (1·4%)	149 (1·6%)	2162 (1·7%)	349 (1·5%)	92 (2·9%)
33–<37 weeks	14 362 (5·7%)	6965 (6·0%)	620 (6·5%)	8643 (6·7%)	1391 (6·0%)	328 (10·3%)
37–43 weeks	226 718 (89·4%)	103 286 (88·9%)	8272 (87·1%)	112 844 (87·9%)	20 672 (88·6%)	2664 (83·3%)
Missing data	8227 (3·2%)	4015 (3·5%)	423 (4·5%)	4238 (3·3%)	876 (3·8%)	90 (2·8%)
**Small for gestational age (<10th centile)**						
No	220 130 (86·8%)	10 0192 (86·2%)	7847 (82·6%)	110 648 (86·2%)	20 049 (85·9%)	2552 (79·8%)
Yes	23 861 (9·4%)	11 295 (9·7%)	1175 (12·4%)	12 831 (10·0%)	2307 (9·9%)	531 (16·6%)
Missing data	9726 (3·8%)	4690 (4·0%)	477 (5·0%)	4955 (3·9%)	989 (4·2%)	115 (3·6%)
**Parity**						
0	112 069 (44·2%)	49 512 (42·6%)	3537 (37·2%)	58 418 (45·5%)	9860 (42·2%)	1335 (41·7%)
≥1	141 167 (55·6%)	66 464 (57·2%)	5947 (62·6%)	69 794 (54·3%)	13 449 (57·6%)	1842 (57·6%)
Missing data	481 (0·2%)	201 (0·2%)	15 (0·2%)	222 (0·2%)	36 (0·2%)	21 (0·7%)
**Multiple births (eg, twins)**						
No or missing data	246 225 (97·0%)	112 631 (96·9%)	9270 (97·6%)	124 576 (97·0%)	22 709 (97·3%)	3110 (97·2%)
Yes	7492 (3·0%)	3546 (3·1%)	229 (2·4%)	3858 (3·0%)	636 (2·7%)	88 (2·8%)
**Birthweight**						
Low (<2500 g)	17 034 (6·7%)	8019 (6·9%)	826 (8·7%)	10 090 (7·9%)	1617 (6·9%)	477 (15·0%)
Normal (2500–3999 g)	198 459 (78·2%)	90 707 (78·1%)	7377 (77·7%)	99 801 (77·7%)	18 109 (77·6%)	2376 (74·3%)
High (≥4000 g)	28 498 (11·2%)	12 761 (11·0%)	819 (8·6%)	13 588 (10·6%)	2630 (11·3%)	230 (7·2%)
Missing data	9726 (3·8%)	4690 (4·0%)	477 (5·0%)	4955 (3·9%)	989 (4·2%)	115 (3·6%)
Congenital anomalies*						
None	242 431 (95·6%)	110 698 (95·3%)	9035 (95·1%)	120 621 (93·9%)	22 088 (94·6%)	2902 (90·7%)
Minor	1713 (0·7%)	874 (0·8%)	71 (0·7%)	1304 (1·0%)	190 (0·8%)	38 (1·2%)
Major	9573 (3·8%)	4605 (4·0%)	393 (4·1%)	6509 (5·1%)	1067 (4·6%)	258 (8·1%)
**Maternal smoking at booking in for birth**						
No	67 184 (26·5%)	24 800 (21·3%)	1653 (17·4%)	31 908 (24·8%)	5406 (23·2%)	568 (17·8%)
Yes	19 991 (7·9%)	8733 (7·5%)	1272 (13·4%)	10 659 (8·3%)	2155 (9·2%)	438 (13·7%)
Missing data	166 542 (65·6%)	82 644 (71·1%)	6574 (69·2%)	85 867 (66·9%)	15 784 (67·6%)	2192 (68·5%)

Data are n (%).

* As classified by the European Congenital Anomalies Registries.

In our cohort, 72 980 (29·5%) infants (age <1 year) lived in a household in which an adult had a mental disorder and 1635 (0·7%) lived in a household with an adult who had had an alcohol-related hospital admission (table 2). These percentages increased with longer duration of follow-up, such that by age 11 years, 19 707 (55·1%) of children had, at some point in their life, lived with an adult who had a mental health diagnosis and 2163 (6·1%) had lived with an adult who had an alcohol-related hospital admission (table 2). A majority of children who were exposed to household mental disorders had lived with an adult who had common mental disorder only (n=114 157, 46·2%); 3139 children (1·2%) had lived with an adult with a psychotic disorder. The incidence of the first emergency hospital admission was highest before age 1 year (33·5 per 100 person-years at risk; table 3).

**Table 2:** Prevalence of mental disorders and alcohol-related hospital admissions in adult household members and single adult households by child's age

	**Born 1998–2012, exposure measured up to age 1 year (n=247 011)**	**Born 1998–2008, exposure measured up to age 4 years (n=157 151)**	**Born 1998–2005, exposure measured up to 7 years (n=97 878)**	**Born 1998–2001, exposure measured up to age 11 years (n=35 744)**
Any household member has ever had a common mental disorder or psychosis diagnosis in GP data	72 980 (29·5%)	65 766 (41·8%)	47 150 (48·2%)	19 707 (55·1%)
Any household member has ever had an alcohol-related hospital admission	1635 (0·7%)	4123 (2·6%)	3906 (4·0%)	2163 (6·1%)
Ever lived in a single adult household (≥16 years old)	54 046 (21·9%)	51 370 (32·7%)	38 088 (38·9%)	15 636 (43·7%)

Data are n (%).

**Table 3:** Number and incidence of first emergency hospital admission by child age groups

	**All causes**	**External causes or injuries**	Victimisation*
**Born 1998–2012 (age 0–<1 year)**			
Total children in age group	253 717	253 717	253 717
Number with first emergency admission (%)	68 758 (27·10%)	3354 (1·32%)	1827 (0·72%)
Total person-years in age group	205 350	248 449	248 634
Incidence (per 100 person-years at risk)	33·48	1·35	0·73
**Born 1998–2011 (age 1–<5 years)**			
Total children in age group	178 520	243 257	244 866
Number with first emergency admission (%)	49 251 (27·59%)	13 522 (5·56%)	948 (0·39%)
Total person-years in age group	178 520	243 257	244 866
Incidence (per 100 person-years at risk)	10·26	1·74	0·12
**Born 1998–2008 (age 5–<8 years)**			
Total children in age group	80 722	145 300	155 635
Number with first emergency admission (%)	6671 (8·26%)	3928 (2·70%)	248 (0·16%)
Total person-years in age group	80 722	145 300	155 635
Incidence (per 100 person-years at risk)	3·50	1·12	0·06
**Born 1998–2005 (age 8–<12 years)**			
Total children in age group	45 831	86 834	95 717
Number with first emergency admission (%)	3221 (7·03%)	2116 (2·44%)	131 (0·14%)
Total person-years in age group	45 831	86 834	95 717
Incidence (per 100 person-years at risk)	2·70	0·91	0·05
**Born 1998–2001 (age 12–<15 years)**			
Total children in age group	14 988	30 176	34 393
Number with first emergency admission (%)	533 (3·56%)	425 (1·41%)	44 (0·13%)
Total person-years in age group	14 988	30 176	34 393
Incidence (per 100 person-years at risk)	2·59	1·03	0·09

* Also includes non-emergency admissions for this cause.

In multivariable analysis, living with an adult household member coded with a mental health disorder was associated with an increased risk of emergency hospital admission, compared with those who had not for all causes (HR 1·17, 95% CI 1·16–1·19), for injuries and external causes (1·14, 1·11–1·18), and for victim-isation (1·55, 1·44–1·67; table 4). These HRs had similar magnitude and direction of effect in univariable analysis (table 4). The magnitude of this increased risk was higher for mothers who had a history of mental disorder before the birth of the baby (HR 1·20, 95% CI 1·18–1·22) or during the first year of life (1·15, 1·13–1·17), compared with mothers who had a mental disorder in later years (appendix p 5). Living with an adult who had a psychotic disorder was associated with an increased risk of emergency admissions for victimisation but not for all causes or injuries or external causes (appendix p 6).

**Table 4:** Regression results from Cox models for time to first emergency hospital admission

	**First all-cause emergency hospital admission**		**First injury or external cause emergency hospital admission**		**First child victimisation hospital admission**	
	Univariable HR (95% CI)	Multivariable HR (95% CI)*	Univariable HR (95% CI)	Multivariable HR (95% CI)*	Univariable HR (95% CI)	Multivariable HR (95% CI)*
**Household member ever had a common mental disorder or psychosis GP code**†						
No	1·00 (ref)	1·00 (ref)	1·00 (ref)	1·00 (ref)	1·00 (ref)	1·00 (ref)
Yes	1·20 (1·19–1·22)	1·17 (1·16–1·19)	1·18 (1·15–1·22)	1·14 (1·11–1·18)	1·73 (1·61–1·86)	1·55 (1·44–1·67)
**Ever any household member with an alcohol-related hospital admission**†						
No	1·00 (ref)	1·00 (ref)	1·00 (ref)	1·00 (ref)	1·00 (ref)	1·00 (ref)
Yes	1·10 (1·02–1·19)	1·01 (0·93–1·09)	1·26 (1·13–1·39)	1·13 (1·01–1·26)	2·05 (1·49–2·82)	1·39 (1·00–1·94)
**Ever in a single parent household**†						
No	1·00 (ref)	1·00 (ref)	1·00 (ref)	1·00 (ref)	1·00 (ref)	1·00 (ref)
Yes	1·07 (1·06–1·08)	1·02 (1·01–1·04)	1·14 (1·10–1·17)	1·05 (1·02–1·08)	1·27 (1·18–1·37)	1·08 (0·99–1·17)
**Townsend deprivation quintile at birth or first 4 months**						
1 (least deprived)	1·00 (ref)	1·00 (ref)	1·00 (ref)	1·00 (ref)	1·00 (ref)	1·00 (ref)
2	1·04 (1·02–1·06)	1·01 (0·99–1·03)	1·15 (1·09–1·20)	1·09 (1·03–1·14)	1·68 (1·44–1·96)	1·47 (1·25–1·72)
3	1·13 (1·11–1·15)	1·06 (1·04–1·09)	1·24 (1·19–1·30)	1·12 (1·07–1·17)	1·77 (1·52–2·06)	1·34 (1·14–1·56)
4	1·17 (1·15–1·19)	1·07 (1·05–1·09)	1·30 (1·24–1·36)	1·13 (1·08–1·18)	2·11 (1·82–2·44)	1·40 (1·21–1·63)
5 (most deprived)	1·26 (1·23–1·28)	1·11 (1·09–1·13)	1·51 (1·45–1·58)	1·22 (1·16–1·28)	3·50 (3·06–4·00)	1·94 (1·68–2·25)
**Sex**						
Male	1·00 (ref)	1·00 (ref)	1·00 (ref)	1·00 (ref)	1·00 (ref)	1·00 (ref)
Female	0·82 (0·81–0·83)	0·83 (0·82–0·83)	0·80 (0·78–0·82)	0·80 (0·78–0·83)	0·93 (0·86–0·99)	0·95 (0·88–1·02)
**Maternal age at childbirth**						
<18 years	1·29 (1·25–1·33)	1·19 (1·15–1·23)	1·67 (1·57–1·78)	1·70 (1·58–1·82)	2·88 (2·48–3·35)	2·30 (1·95–2·71)
18–24 years	1·17 (1·16–1·19)	1·13 (1·11–1·14)	1·32 (1·28–1·36)	1·28 (1·24–1·33)	1·80 (1·64–1·97)	1·51 (1·37–1·65)
25–29 years	1·00 (ref)	1·00 (ref)	1·00 (ref)	1·00 (ref)	1·00 (ref)	1·00 (ref)
30–34 years	0·89 (0·88–0·90)	0·92 (0·90–0·93)	0·86 (0·83–0·89)	0·88 (0·85–0·91)	0·75 (0·67–0·84)	0·86 (0·77–0·97)
≥35 years	0·86 (0·85–0·88)	0·89 (0·87–0·91)	0·86 (0·82–0·90)	0·87 (0·83–0·92)	0·82 (0·72–0·93)	0·96 (0·84–1·10)
**Gestational age at birth**						
24–<28 weeks (extremely preterm)	2·73 (2·51–2·97)	2·50 (2·30–2·72)	1·00 (0·77–1·30)	0·95 (0·73–1·23)	2·70 (1·81–4·04)	2·25 (1·49–3·38)
28–<3 weeks (very preterm)	1·80 (1·73–1·88)	1·72 (1·65–1·80)	1·21 (1·09–1·35)	1·16 (1·04–1·30)	2·45 (1·99–3·01)	2·13 (1·72–2·63)
33–<37 weeks (moderately preterm)	1·42 (1·39–1·45)	1·39 (1·36–1·42)	1·07 (1·01–1·13)	1·05 (0·99–1·11)	1·96 (1·75–2·20)	1·83 (1·62–2·06)
≥37 weeks (term)	1·00 (ref)	1·00 (ref)	1·00 (ref)	1·00 (ref)	1·00 (ref)	1·00 (ref)
**Small for gestational age (<10th centile for gestation and sex-specific birthweight)**						
No	1·00 (ref)	1·00 (ref)	1·00 (ref)	1·00 (ref)	1·00 (ref)	1·00 (ref)
Yes	1·13 (1·11–1·15)	1·07 (1·05–1·09)	1·07 (1·03–1·12)	1·01 (0·97–1·06)	1·95 (1·78–2·14)	1·54 (1·39–1·69)
**Breastfeeding at birth or 6–8 weeks**						
No	1·00 (ref)	1·00 (ref)	1·00 (ref)	1·00 (ref)	1·00 (ref)	1·00 (ref)
Yes	0·87 (0·86–0·88)	0·94 (0·93–0·95)	0·83 (0·81–0·86)	0·96 (0·93–0·99)	0·59 (0·54–0·63)	0·89 (0·81–0·97)
**Parity**						
0	1·00 (ref)	1·00 (ref)	1·00 (ref)	1·00 (ref)	1·00 (ref)	1·00 (ref)
≥1	0·91 (0·90–0·92)	0·96 (0·95–0·97)	1·06 (1·04–1·09)	1·18 (1·15–1·21)	1·08 (1·01–1·16)	1·27 (1·17–1·37)
**Multiple births (eg, twins)**						
No or no answer	1·00 (ref)	1·00 (ref)	1·00 (ref)	1·00 (ref)	1·00 (ref)	1·00 (ref)
Yes	1·07 (1·04–1·10)	0·90 (0·87–0·93)	0·93 (0·86–1·01)	0·98 (0·90–1·06)	0·94 (0·76–1·16)	0·73 (0·58–0·91)
**Congenital anomalies**						
None	1·00 (ref)	1·00 (ref)	1·00 (ref)	1·00 (ref)	1·00 (ref)	1·00 (ref)
Major or minor	1·90 (1·86–1·94)	1·81 (1·77–1·85)	1·20 (1·14–1·28)	1·18 (1·11–1·25)	2·19 (1·94–2·46)	1·93 (1·71–2·19)
**Maternal cigarette smoking at booking in**						
No	1·00 (ref)	1·00 (ref)	1·00 (ref)	1·00 (ref)	1·00 (ref)	1·00 (ref)
Yes	1·18 (1·16–1·19)	1·06 (1·03–1·08)	1·32 (1·27–1·36)	1·13 (1·09–1·17)	3·08 (2·61–3·64)	2·17 (1·78–2·65)

* Adjusted for all variables in the table.

† For children aged 0–12 years. HR=hazard ratio.

Compared to those not exposed, children with a household member who had an alcohol-related hospital admission had a higher risk of emergency admissions for injuries and external causes (HR 1·13, 95% CI 1·01–1·26), and victimisation (1·39, 1·00–1·94) in the multivariable model. Although there was an increased risk for all cause of emergency admissions in univariable analysis, this was not statistically significant in the multivariable model. These effects did not vary with the timing of this exposure (appendix p 5). We repeated this analysis using the Andersen-Gill model and obtained nearly identical HRs, probably because most emergency admissions occur during the first year of life (data not shown).

Greater social deprivation, maternal age younger than 18 years, and living in a single adult household had a residual increased risk of emergency hospital admissions for all causes and injuries and external causes (table 4). Emergency admissions because of victimisation were associated with greater social deprivation and maternal age younger than 18 years (table 4). Table 5 shows how the risk of emergency hospital admissions increases with linear combinations of each exposure, measures of deprivation, and family structure. For example, compared with children who lived in the least deprived quintile who were not exposed to household mental health or alcohol misuse, children who were born to young mothers who smoked during pregnancy and lived in an area with the highest level of deprivation had an increased risk of all cause emergency hospital admission (HR 1·40 95% CI 1·35, 1·46, table 5). This risk was even higher if they also lived with an adult with a mental disorder (table 5).

**Table 5:** Risk of emergency admission for all causes associated with combinations of exposures and sociodemographic characteristics

	**First all-cause emergency hospital admission**	**First injury or external cause emergency hospital admission**	**First child victimisation hospital admission**
Most deprived quintile at birth or first 4 months AND maternal age at childbirth <18 years	1·32 (1·28–1·38)	2·07 (1·91–2·24)	4·47 (3·60–5·55)
Most deprived quintile at birth or first 4 months AND maternal age at childbirth <18 years AND maternal smoking at booking in for birth	1·40 (1·35–1·46)	2·34 (2·16–2·54)	9·70 (7·68–12·26)
Most deprived quintile at birth or first 4 months AND maternal age at childbirth <18 years AND household member ever had a mental disorder	1·55 (1·49–1·61)	2·37 (2·17–2·57)	6·93 (5·52–8·70)
Most deprived quintile at birth or first 4 months AND maternal age at childbirth <18 years AND maternal smoking at booking in for birth AND household member ever had a mental disorder	1·64 (1·58–1·71)	2·68 (2·46–2·92)	15·04 (11·78–19·20)
Most deprived quintile at birth or first 4 months AND maternal age at childbirth <18 years AND maternal smoking at booking in for birth AND household member ever had an alcohol-related hospital admission	1·41 (1·29–1·54)	2·64 (2·32–3·02)	13·53 (9·09–20·14)
Most deprived quintile at birth or first 4 months AND maternal age at childbirth <18 years AND maternal smoking at booking in for birth AND household member ever had a mental disorder AND household member ever had an alcohol-related hospital admission	1·65 (1·51–1·81)	3·02 (2·64–3·46)	20·97 (14·03–31·36)

Data are hazard ratio (95% CI). Combinations based on linear combinations of adjusted model in table 4. Reference category: least deprived quintile of area-level deprivation, maternal age at childbirth was 25–29 years, no maternal smoking at booking in for birth, no exposure to household mental disorder or alcohol-related hospital admission.

## Discussion

We found that living with a household member who had a mental disorder was associated with increased risks of emergency hospital admissions in children for all causes, injuries, external causes, and victimisation, and this risk was greater if the child was born to a young mother or lived in a deprived area. Living with an adult who had an alcohol-related hospital admission was associated with an increased risk of emergency admissions because of injuries, external causes, and victimisation but not all causes.

Many studies have found associations between adverse childhood experiences, and mental disorders and substance misuse in adulthood,^1^ early initiation of alcohol use, binge drinking, and heavy episodic drinking during adolescence,^28^ but none have investigated emergency hospital admissions during childhood. Some studies were based on surveys of adults, requiring self-report of exposure to adverse childhood experiences and simultaneous reporting of outcomes of interest, which could be subject to recall and common source bias. Our study is the first, to our knowledge, to use objectively measured indicators of household mental disorder and alcohol misuse, and explore their association with emergency hospital admissions during childhood. We chose this as our primary outcome measure because children and young people are vulnerable and dependent on their carers and emergency hospital admissions are an indicator of physical morbidity and unmet need.

In our study, one in three households with infants had an adult with a mental disorder. This proportion is more than double the estimate from another study,^29^ which asked adults aged 16–65 years if they had lived with anyone who was depressed, mentally ill, or suicidal when they were growing up. This lower prevalence might be subject to recall bias or participants being more likely to be aware of and report severe mental illnesses. Common mental disorders affect 16% of the population,^4^ with higher prevalence in more deprived populations. These disorders do not usually affect insight and cognition but can cause emotional distress and interfere with daily functioning. Some studies have estimated up to a quarter of pregnant women experience some form of psychosocial stress, anxiety, or depressive symptoms.^30^ Such disorders are associated with adverse birth outcomes, conduct problems, and infections during childhood.^11^, ^12^, ^30^

Our measure of household mental health exposure included both the more prevalent common mental disorders and severe mental illnesses that affect smaller numbers of the population.^2^, ^3^, ^4^ We were able to assess specifically the risk of emergency admissions in children associated with exposure to psychotic disorders in the household, but lacked precision in this estimate because of the relatively small number of children in the cohort who had this exposure. Despite our wide definition, we showed an important and sizeable effect of household mental disorders on emergency hospital admissions during childhood. This finding suggests that the less severe forms of mental disorders affecting a larger number of adults can have significant effects on the health of children. This effect could be due to the impact of mental illness on family relationships and disrupted routines leading to inconsistent care, although we were not able to explore this possibility in our study.

Our measure of alcohol misuse in the household was based on alcohol-related hospital admissions, which included only the most severe cases and was therefore probably an underestimate of alcohol-related problems in the general population. Studies of the effects of parental alcohol misuse have shown increased risk in children of alcohol misuse during adolescence, substance misuse, behavioural problems, and impaired mental health,^7^ but have not sufficiently explored physical health outcomes in children.^7^, ^9^ Alcohol misuse in a family can lead to financial difficulties, inability to meet children's needs, and lack of supervision leading to children witnessing violence and being shouted at or physically hurt.^9^, ^31^ We showed that children living with an adult who had an alcohol-related hospital admission had a 13% increased risk of emergency admissions for injuries and a 44% increased risk of emergency admissions for victimisation, compared with children who were unexposed. However, household mental illness, alcohol misuse, and emergency admissions are each associated with social disadvantage. Although we adjusted our results for deprivation and some markers of social disadvantage such as young maternal age at childbirth and cigarette smoking, it is possible that the associations we report were subject to residual or unmeasured confounding. Overall, our results suggest that minimising harm to children requires actions to address the socioeconomic conditions and other factors that imply vulnerability in families such as young maternal age and cigarette smoking during pregnancy, mental disorders and alcohol misuse, which all appear to contribute cumulatively to risk of hospital admissions.

The main strengths of this study are its large number of unselected participants from a total population cohort, longitudinal follow-up over 14 years, and statistical analysis that took account of the temporal relationship between exposures and outcomes and objective measurement of exposures using routine health-care data. Further, we were able to ascertain the effect of combinations of exposures while allowing for differences in the magnitude of effect associated with each exposure. However, several adverse childhood events could not be measured with routine data, such as domestic violence, and sexual and physical abuse. These exposures are more severe but less common. Our measure of mental disorder in the household combined common mental disorders and less prevalent psychotic disorders. We assessed separately the effect of exposure to psychotic disorders but the common mental disorder category includes symptoms and diagnoses with a range of severity. As a result, there might have been a low threshold for being coded positively, which may have attenuated the effect of mental disorders, such that the true effect may be greater than what we have estimated. Household alcohol-related hospital admissions were rare (0·7%), and about a third of these were due to alcohol use disorder, and therefore likely to be an underestimate of the population prevalence of alcohol-related disorders. Linkage of other datasets, such as those from general practice would provide broader measurement of alcohol-related problems. Further, we were not able to determine family relationships within households and therefore could not distinguish maternal or paternal exposures and their effects. The use of routine data for epidemiological analyses can be subject to misclassification because of the coding process. Our use of validated algorithms for ascertaining mental disorders^16^, ^17^ reduced this risk; any misclassification is likely to be non-differential and bias the results towards the null. We required GP data to code mental disorders and had to exclude children who lived with adults for whom these data were not available. However, we considered the risk of selection bias to be low because two in three GP practices in Wales contribute data, and the distribution of sociodemographic characteristics (including area-level deprivation) in our cohort was similar to that of the Welsh population.

Alcohol misuse and mental disorders are a substantial burden. One in three households with infants include an adult with poor mental health, and nearly one in ten adults have hazardous or higher levels of alcohol consumption. The higher risk of emergency admissions in children associated with these exposures supports the need for policy measures to identify and provide support to young families that are affected, in addition to addressing the socioeconomic conditions that are associated with these exposures and their consequences.
