# Supplementary material for: Risk of emergency hospital admission in children associated with mental disorders and alcohol misuse in the household: an electronic birth cohort study
Source: Lancet Public Health. 2018 Jun 5;3(6):e279–88. doi: 10.1016/S2468-2667(18)30069-0 (PMC5990492; doi:10.1016/S2468-2667(18)30069-0)
Supplement: Supplementary appendix [file mmc1.pdf]

THE LANCET
Public Health

Supplementary appendix

This appendix formed part of the original submission and has been peer reviewed. We post it as supplied by the authors.

Supplement to: Paranjothy S, Evans A, Bandyopadhyay A, et al. Risk of emergency hospital admission in children associated with mental disorders and alcohol misuse in the household: an electronic birth cohort study. *Lancet Public Health* 2018; published online May 15. http://dx.doi.org/10.1016/S2468-2667(18)30069-0.

Appendix: Supplementary tables

Supplementary table 1: WECC Data sources

Data source	Description
Public Health Birth files from the Office for National Statistics (ONSB) - from 2003	Data on all births in Wales or to mothers who are usually resident in Wales
National Community Child Health Database (NCCHD) - from 1987	A national database of all children resident in Wales or born in a Welsh hospital, containing data collected at birth such as parity, mode of delivery, gestation, birth weight, gender, breastfeeding, and Apgar Score
Public Health Mortality Files from the Office for National Statistics (ONSM) - from 2002	Data on all deaths in Wales or of individuals who are usually resident in Wales
Patient Episode Dataset for Wales (PEDW) - from 1998	Demographic and clinical data on all inpatient and day-case admissions in National Health Service Wales hospitals and all Welsh residents treated in other UK countries
All Wales Perinatal Survey (AWPS) -from 1993	A database of perinatal and infant mortality in Wales including infants from 20 weeks' gestation to 1 year of age, who die in a Welsh hospital or whose mother is usually resident in Wales
Congenital Anomaly Register and Information Service (CARIS) - from 1998	A population-based register of any foetus or infant who has a congenital anomaly whose mother is usually resident in Wales at the time of birth; congenital anomalies are defined by the European network of population-based registries for the epidemiologic surveillance of congenital anomalies

Supplementary figure 1: Direct Acyclic Graph to inform variable selection

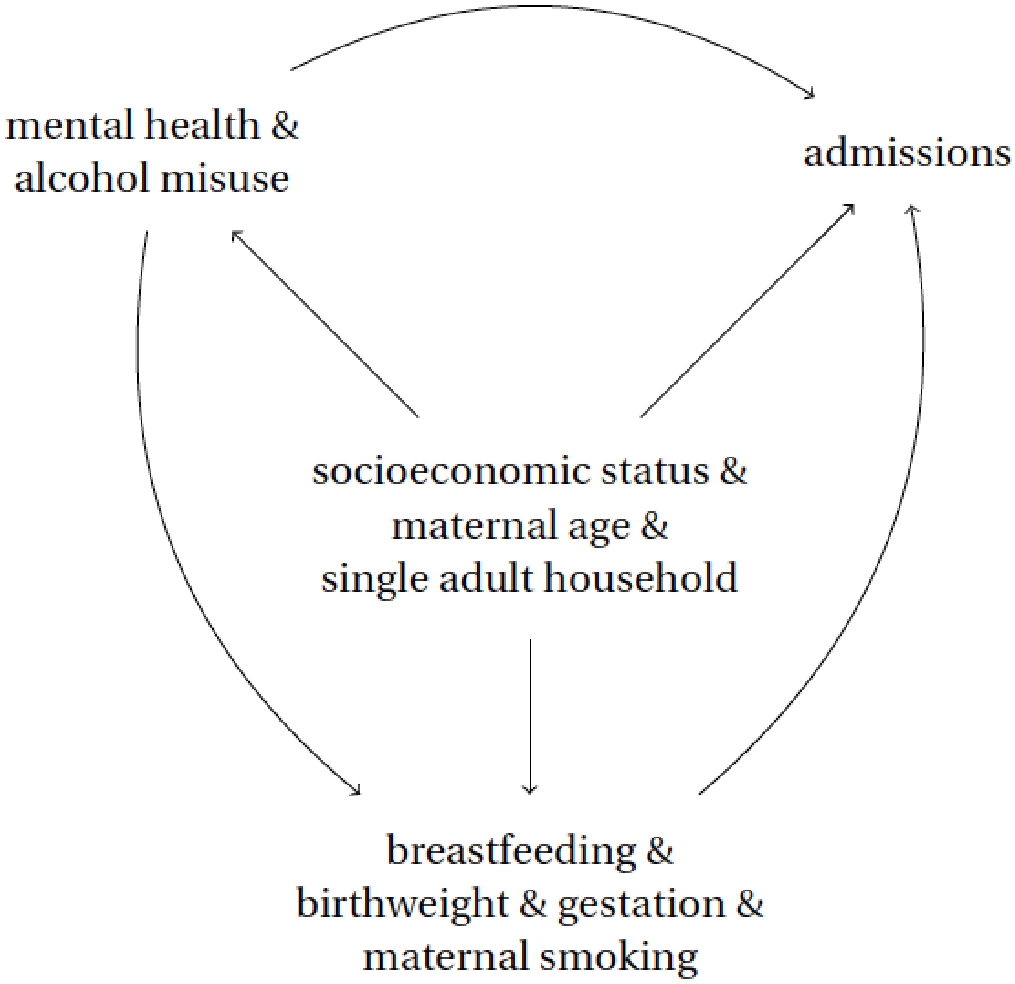

Supplementary table 2: Social-demographic data for Wales

	Total	
	**n**	**(%)**
Townsend Deprivation quintile: from 2003, child's age 0 – 14 years old[a]		
1 - least deprived	-	(19.3)
2	-	(19.3)
3	-	(19.3)
4	-	(20.5)
5 - most deprived	-	(21.7)
Sex: from 2001, child's age 0 – 14 years old[b]		
Male	281767	(51.3)
Female	267437	(48.7)
Breastfeeding at birth: Welsh residents 2011[c]		
No	14469	(40.5)
Yes	18062	(50.6)
no answer	3151	(8.8)
Maternal age at childbirth: Welsh residents 2011 [c]		
<16	57	(0.2)
16-19	2409	(6.8)
19-24	8115	(22.7)
25-29 years	10268	(28.8)
30-34	9107	(25.5)
35+	5722	(16.0)
no answer	4	(0.01)
Gestational age at birth: Welsh residents 2011 [c]		
20-<32 weeks	443	(1.2)
32-<37 weeks	2094	(5.9)
37-43 weeks	32985	(92.4)
no answer	160	(0.4)
Birthweight: Welsh residents 2011 [c]		
Low: < 2500g	2403	(6.7)
Normal: ≥ 2500 - < 4000g	28991	(81.2)
High: ≥ 4000g	4249	(11.9)
no answer	39	(0.1)

[a] Deprivation and health – report for the National Public Health Service for Wales 2004; [b] Welsh data from the UK Census 2001 at https://statswales.gov.wales/; [c] Births in Wales 2001 - 2011: Data from the National Community Child Health Database 2012.

Supplementary table 3: Complete case regression results from Cox models for time to first emergency hospital admission

	First all-cause emergency hospital admission		First injury or external cause emergency hospital admission		First child victimisation hospital admission	
	Multivariable[a]		Multivariable[a]		Multivariable[a]	
N=78,207	HR[b]	95% CI	HR[b]	95% CI	HR[b]	95% CI
Household member ehad a common mental disorder /psychosis GP code: for child pre-birth - 12 years.						
No	1.00		1.00		1.00	
Yes	1.14	(1.11-1.17)	1.15	(1.09-1.21)	1.16	(1.00-1.35)
Ever in a single parent household: for child from birth - 12 years.						
No	1.00		1.00		1.00	
Yes	1.02	(1.00-1.04)	1.09	(1.03-1.15)	1.08	(0.93-1.26)
Ever any household member with an alcohol-related hospital admission: for child from birth - 12 years.						
No	1.00		1.00		1.00	
Yes	0.91	(0.77-1.06)	1.02	(0.82-1.27)	0.56	(0.18-1.76)
Townsend deprivation quintile at birth / first 4 months						
1 - least deprived	1.00		1.00		1.00	
2	0.99	(0.96-1.03)	1.10	(1.00-1.21)	1.14	(0.89-1.47)
3	1.05	(1.01-1.08)	1.24	(1.14-1.36)	0.93	(0.72-1.19)
4	1.08	(1.04-1.12)	1.23	(1.12-1.34)	0.95	(0.74-1.22)
5 - Most deprived	1.09	(1.05-1.13)	1.32	(1.21-1.45)	1.23	(0.96-1.57)
Sex						
Male	1.00		1.00		1.00	
Female	0.82	(0.81-0.84)	0.82	(0.78-0.86)	0.87	(0.76-1.00)
Maternal age at childbirth						
<18	1.24	(1.16-1.32)	1.70	(1.49-1.94)	1.77	(1.26-2.48)
18-24	1.14	(1.11-1.18)	1.28	(1.20-1.37)	1.28	(1.07-1.52)
25-29 years old	1.00		1.00		1.00	
30-34	0.92	(0.89-0.94)	0.92	(0.85-0.98)	0.84	(0.69-1.03)
35+	0.87	(0.84-0.90)	0.90	(0.83-0.98)	0.86	(0.68-1.09)
Gestational age at birth						
24 - < 28 weeks: extremely preterm	3.16	(2.53-3.95)	0.54	(0.22-1.29)	0.90	(0.13-6.42)
28 - < 33 weeks: v preterm	1.90	(1.74-2.07)	1.09	(0.86-1.37)	2.42	(1.56-3.75)
33 - < 37 weeks: moderately preterm	1.44	(1.38-1.50)	1.00	(0.89-1.11)	1.57	(1.23-2.02)
37+ weeks: term	1.00		1.00		1.00	
Small for gestational age (<10th centile for gestation and gender-specific birthweight)						
No	1.00		1.00		1.00	
Yes	1.09	(1.05-1.12)	1.03	(0.95-1.11)	1.35	(1.12-1.63)
Breastfeeding at birth / 6-8 weeks						
No	1.00		1.00		1.00	
Yes	0.94	(0.92-0.96)	1.02	(0.97-1.08)	1.12	(0.98-1.29)
Parity						
No	1.00		1.00		1.00	
Yes	0.96	(0.94-0.98)	1.16	(1.10-1.22)	1.10	(0.95-1.27)
Multiple births (e.g. twins)						
No/no answer	1.00		1.00		1.00	
Yes	0.89	(0.84-0.95)	1.04	(0.89-1.22)	0.57	(0.35-0.94)
Congenital anomalies						
None	1.00		1.00		1.00	
Major/minor	1.91	(1.82-2.00)	1.22	(1.09-1.37)	1.95	(1.52-2.51)
Maternal cigarette smoking at booking in						
No	1.00		1.00		1.00	
Yes	1.06	(1.04-1.09)	1.13	(1.07-1.20)	2.18	(1.89-2.52)

[a] adjusted for all variables in the model; [b] hazard ratio.

Supplementary table 4: Timing of exposure and risk of emergency hospital admission

	First all-cause emergency hospital admission	
	Multivariable[a]	
	HR[b]	95% CI
History of mental disorder in household member before birth of the child		
No	1.00	
Yes	1.20	(1.18-1.22)
Mental disorder in household member from birth to age <1 year		
No	1.00	
Yes	1.15	(1.13-1.17)
Mental disorder in household member from age 1 - < 5 years		
No	1.00	
Yes	1.15	(1.09-1.20)
Mental disorder in household member from age 5 - < 8 years		
No	1.00	
Yes	1.12	(1.05-1.21)
Mental disorder in household member from age 8 - < 12 years		
No	1.00	
Yes	1.16	(0.97-1.38)

[a] adjusted for Townsend deprivation quintile, sex, maternal age at childbirth, gestational age at birth, small for gestational age (<10th centile), breastfeeding at 6-8 weeks, parity, multiple births, congenital anomalies, maternal cigarette smoking at booking in for pregnancy; [b] hazard ratio.

	First all-cause emergency hospital admission	
	Multivariable[a]	
	HR[b]	95% CI
Alcohol related hospital admission in household member from birth to age <1 year		
No	1.00	
Yes	1.00	(0.89-1.11)
Alcohol related hospital admission in household member from age 1 - < 5 years		
No	1.00	
Yes	1.09	(0.93-1.27)
Alcohol related hospital admission in household member from age 5 - < 8 years		
No	1.00	
Yes	1.03	(0.85-1.25)
Alcohol related hospital admission in household member from age 8 - < 12 years		
No	1.00	
Yes	1.13	(0.78-1.65)

[a] adjusted for Townsend deprivation quintile, sex, maternal age at childbirth, gestational age at birth, small for gestational age (<10th centile), breastfeeding at 6-8 weeks, parity, multiple births, congenital anomalies, maternal cigarette smoking at booking in for pregnancy; [b] hazard ratio.

Supplementary table 5: Regression results from Cox models for time to first emergency hospital admission associated with common mental and psychotic disorders.

	First all-cause emergency hospital admission		First injury or external cause emergency hospital admission		First child victimisation hospital admission	
	Multivariable[a]		Multivariable[a]		Multivariable[a]	
	HR[b]	95% CI	HR[b]	95% CI	HR[b]	95% CI
Household member ever had a common mental disorder GP code: for child pre-birth - 12 years.						
No	1.00		1.00		1.00	
Yes	1.17	(1.16–1.19)	1.14	(1.11–1.18)	1.52	(1.41–1.64)
Household member ever had a psychosis GP code: for child pre-birth - 12 years.						
No	1.00		1.00		1.00	
Yes	0.99	(0.93–1.06)	1.08	(0.95–1.24)	1.78	(1.37–2.32)

[a] adjusted for ever any household member with an alcohol-related hospital admission, ever in a single adult household, deprivation quintile at birth/first 4 months, sex, maternal age at childbirth, gestational age at birth, small for gestational age (<10th centile for gestation and gender-specific birthweight), breastfeeding at birth / 6-8 weeks, parity, multiple births (e.g. twins), congenital anomalies, maternal cigarette smoking at booking for birth; [b] hazard ratio.
